# Integrated Modeling Approach for Electrochemical Regeneration of Alkaline CO_2_ Capture Solvents

**DOI:** 10.1021/acs.iecr.5c00497

**Published:** 2025-05-08

**Authors:** Fariborz Shaahmadi, Katia Piscina, Sotirios Efstathios Antonoudis, Qingdian Shu, Sara Vallejo Castaño, Grigorios Itskos, Konstantinos Atsonios, Susana Garcia, Mijndert van der Spek

**Affiliations:** † Research Centre for Carbon Solutions, School of Engineering and Physical Sciences, 150983 Heriot-Watt University, Edinburgh EH14 4AS, U.K.; ‡ 54576 Centre for Research & Technology Hellas/Chemical Process and Energy Resources Institute (CERTH/CPERI), GR 57001 Thermi, Greece; § 361182 Wetsus, European Centre of Excellence for Sustainable Water Technology, 8911 MA Leeuwarden, Netherlands

## Abstract

This work introduces a model for electrochemical CO_2_ capture and solvent regeneration integrated with an Aspen Plus flowsheet. The model is built in Aspen Custom Modeler and designed to seamlessly integrate with ASPEN Plus software, allowing for comprehensive simulation of the CO_2_ capture process using an electrochemical cell to regenerate the solvent. The model includes detailed descriptions of the mass and energy balances in the electrochemical stack compartments, mass transport over the ion exchange membrane, and potential losses through the stack. The validity of the model was assessed against laboratory measurements. The model was exemplified by modeling a CO_2_ capture pilot plant for three different flue gases, from cement, magnesite, and gas-fired CHP production. The results of the integrated absorber-electrochemical system reveal key trade-offs among CO_2_ capture efficiency, energy consumption, and throughput, highlighting the performance differences across the three case studies.

## Graphical abstract

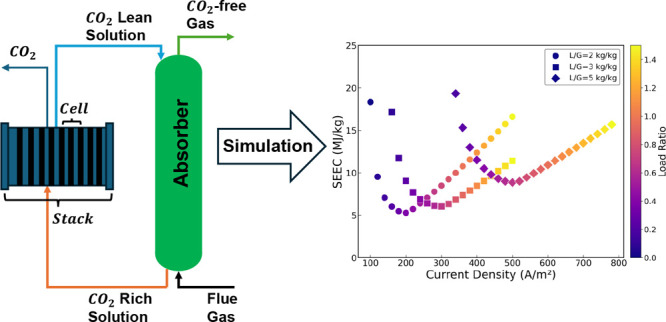

## 1 Introduction

The significant increase in the global average surface temperature, which has exceeded 1.5 °C since preindustrial times, underscores the urgent need to address anthropogenic climate change.^1^ This warming trend has severe environmental and socio-economic implications, emphasizing the necessity of innovative strategies to reduce carbon emissions. Among these, CO_2_ capture technologies play a key role, and research is increasingly focused on improving efficiency, scalability, and sustainability.^2–8^

Postcombustion CO_2_ capture, particularly using amine-based absorbents, remains the most widely adopted method due to its high capture efficiency and compatibility with existing industrial infrastructure.^9^ Recent advances have reduced the thermal energy demand of amine systems to below 2.5 GJ/t CO_2_.^10^ However, persistent challenges such as solvent volatility, degradation, and the release of harmful byproducts underscore the need for alternative approaches.^11^ Alkaline solvents, especially potassium hydroxide (KOH), present a promising alternative. Their stability, nonvolatility, and ability to react with CO_2_ to form bicarbonate and carbonate compounds make them an environmentally attractive option.^12^ Unlike amine-based systems, KOH solutions avoid the production of harmful degradation products. Furthermore, electrochemical regeneration of KOH-based systems offers a pathway to greater sustainability by using renewable electricity, aligning with the transition to a carbon-neutral future.^13^

Electrochemical regeneration technologies have gained traction for their potential to overcome energy and operational limitations. These systems employ a pH-swing mechanism, where CO_2_ is released in low-pH zones and the solvent is regenerated in high-pH zones, enabling subsequent desorption and regeneration.^14^ While existing commercial process modeling platforms excel at simulating absorption processes, their inability to accurately represent electrochemical regeneration hinders comprehensive carbon capture system design. Computational modeling will be instrumental in designing and optimizing these processes, shedding light on critical factors such as electrode configurations, overpotentials, and faradaic efficiency.^15^ Despite the advancements, significant research gaps remain, particularly regarding upscaling, durability, and energy demands in real-world applications, as well as long-term performance under continuous operation.^16^

The ConsenCUS project addresses these challenges by integrating KOH-based solvents with electrochemical regeneration, upscaling, and demonstrating this technology at TRL7. This approach utilizes widely available bipolar membrane electrodialysis (BPMED) technology and pH-gradient mechanisms for solvent regeneration and CO_2_ desorption.^17^ We introduce a computational model—developed in Aspen Plus and Aspen Custom Modeler—that integrates CO_2_ absorption and electrochemical regeneration into a single, cohesive workflow. This first-of-its-kind model integrates thermodynamic equilibrium calculations to allow integrated absorber-electrochemical regeneration systems design and optimization in a computationally affordable manner. Key advancements include mapping regeneration efficiency and energy consumption across variable current densities, and validation of system scalability. The model is tested using flue gases from three industrial case studies: cement, magnesite, and natural gas-fired combined heat and power production. The resulting model is openly accessible via [https://github.com/mijndertvanderspek/ConsenCUS].

## 2 Process Overview

The CO_2_ capture process combines alkaline absorption using potassium hydroxide (here, 1 M KOH) with electrochemical absorbent regeneration as shown in Figure 1. KOH was selected due to its strong reactivity with CO_2_ and its ability to form stable potassium carbonate (K_2_CO_3_) and potassium bicarbonate (KHCO_3_) compounds during absorption, additional to its negligible volatility and absent oxidative degradation.

**Figure 1:**
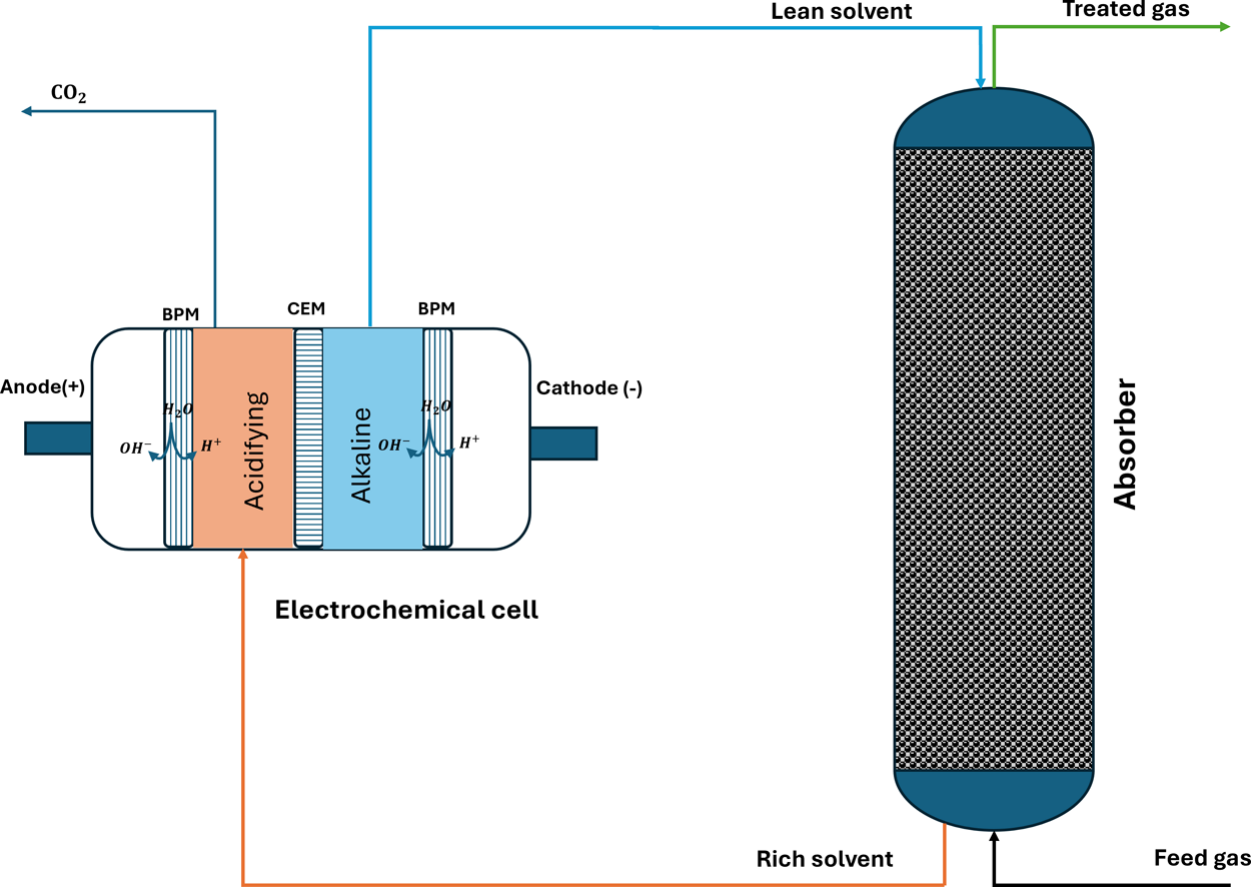
Schematic of the CO_2_ capture process.

The absorption mechanism relies on the following key equilibrium and kinetic reactions:

$${\mathrm{CO}}_{2}+{\mathrm{OH}}^{-}⇌{{\mathrm{HCO}}_{3}}^{-}$$ 1

$${\mathrm{CO}}_{2}+2H_{2}O⇌{{\mathrm{HCO}}_{3}}^{-}+H_{3}O^{+}$$ 2

$${{\mathrm{HCO}}_{3}}^{-}+{\mathrm{OH}}^{-}⇌{{\mathrm{CO}}_{3}}^{2-}+H_{2}O$$ 3

$$\mathrm{KOH}⇌K^{+}+{\mathrm{OH}}^{-}$$ 4

The pilot plant absorber column has an effective packing height of 9 m and a diameter of 0.6 m. Flue gas is fed to the absorber after treatment in a direct contact cooler (DCC) to remove water, polish the sulfur content, and reduce the temperature. The flue is countercurrently contacted with the KOH solution, equal to amine-based absorption, producing a rich absorbent flow leaving the bottom of the column, while CO_2_ lean flue gas is exhausted via the absorber top.

Flue gases from three industrial sources were analyzed: cement production, natural-gas fired combined heat and power (CHP) production, and magnesite processing. These flue gases contain 13.5, 3.5, and 9.1%, CO_2_, respectively.

### 2.1 Electrochemical Regeneration

The electrochemical regeneration process facilitates CO_2_ desorption by transferring the CO_2_-loaded solution into an electrochemical cell, where a pH swing, driven by electrochemical reactions, enables the release of CO_2_. The electrochemical cell consists of two compartments: an acidifying compartment and an alkaline compartment, separated by a cation exchange membrane (CEM) as shown in Figure 2. A bipolar membrane (BPM) facilitates the pH swing and drives the and electrodialysis process, by dissociating water into protons (H^+^) and hydroxide ions (OH^–^), as detailed below.

$$H_{2}O\overset{K_{w}}{⇌}H^{+}+{\mathrm{OH}}^{-}$$ 5

**Figure 2:**
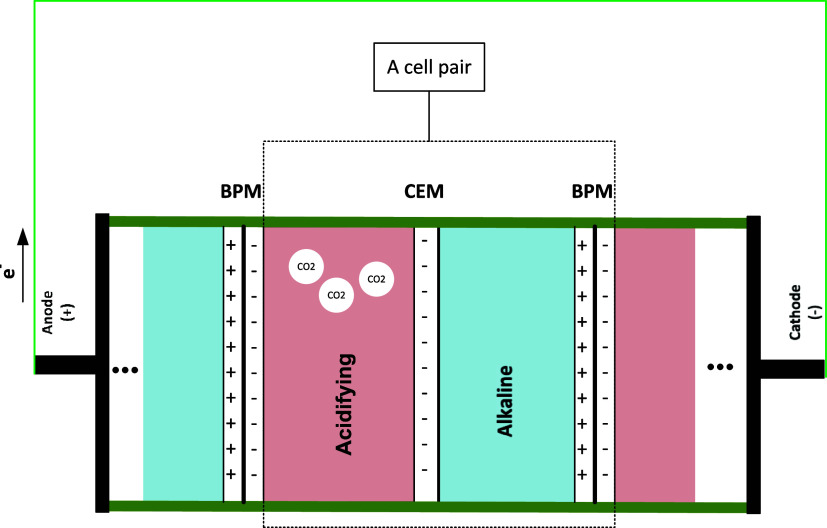
Schematic of the electrochemical cell used for CO_2_ desorption and solvent regeneration.

At the two ends of the electrochemical stacks, an anode and cathode facilitate the flow of electrons into and out of the assembly. At the anode, water is oxidized to produce protons and oxygen gas (O_2_), while at the cathode, water is reduced to produce hydroxide ions and hydrogen gas (H_2_):

$$\mathrm{Anode}:2H_{2}O\to O_{2}+4H^{+}+4e^{-}$$ 6

$$\mathrm{Cathode}:4H_{2}O+4e^{-}\to 2H_{2}+4{\mathrm{OH}}^{-}$$ 7

The protons (H^+^) produced at the anode react with the bicarbonate (HCO_3_^–^) and carbonate ions (CO_3_^2–^) in the CO_2_-loaded solution, leading to CO_2_ desorption via the formation of unstable carbonic acid. The CO_2_ is released from the solution as follows:

$${\mathrm{CO}}_{2}(\mathrm{aq})+H_{2}O\overset{K_{1}}{⇌}{{\mathrm{HCO}}_{3}}^{-}+H^{+}$$ 8

$${{\mathrm{HCO}}_{3}}^{-}\overset{K_{2}}{⇌}{{\mathrm{CO}}_{3}}^{2-}+H^{+}$$ 9

The equilibrium constants (*K*_1_, *K*_2_, and *K*_w_) were obtained from literature sources^18^. These constants play a crucial role in describing the equilibrium behavior of CO_2_ in aqueous solutions and in determining the CO_2_ desorption efficiency during the electrochemical regeneration process. The produced carbonic acid/aqueous CO_2_ evolves from the liquid to form gaseous CO_2_, that can be separated from the acidifying chamber outlet flow, e.g., in a flash vessel of membrane contactor.

The reaction from (bi)­carbonate to carbonic acid and CO_2_ and the surplus of protons in the acidifying chamber generate a flow of potassium ions (plus protons) across the CEM, where it recombines with the hydroxide formed at the alkaline side of the next BPM.

The electrochemical cell effectively uses the pH swing mechanism to achieve both solvent regeneration and CO_2_ desorption, thereby enhancing the overall sustainability of the CO_2_ capture process.

The ConsenCUS pilot plant utilizes two electrochemical stacks (126 cell pairs each) for solvent regeneration, operating in parallel mode (for increased flow) or series mode (for enhanced voltage). Each stack features cell pairs with 0.1936 m^2^ effective area per cell. More details of the ConsenCUS pilot plant are provided in Table 3.

## 3 Model Development

### 3.1 Absorber Modeling

The CO_2_ capture section was simulated in Aspen Plus, using the Electrolyte-NRTL (elecNRTL) model to describe the nonideal behavior of electrolyte mixtures. The E-NRTL model was selected based on its capability to accurately represent the interactions between CO_2_, KOH, water, and the resulting ionic species in aqueous solutions. The binary parameters of the model were sourced from existing literature. The absorption was modeled as a rate-based process. The kinetic reactions governing CO_2_ absorption in alkaline media follow a second-order rate law, distinct from equilibrium pathways, and we here explicitly modeled the hydroxide and water pathways for CO_2_ hydrolysis, to better capture the different kinetic regimes across the observed pH ranges in the absorber:

$${\mathrm{CO}}_{2}+{\mathrm{OH}}^{-}\overset{k_{1}}{\to }{{\mathrm{HCO}}_{3}}^{-},\;\mathrm{rate}=k_{1}[{\mathrm{CO}}_{2}][{\mathrm{OH}}^{-}]$$ 10

$${\mathrm{CO}}_{2}+2H_{2}O\overset{k_{2}}{\to }{{\mathrm{HCO}}_{3}}^{-}+H_{3}O^{+},\;\mathrm{rate}=k_{2}[{\mathrm{CO}}_{2}][H_{2}O]$$ 11

These rate laws were implemented using the experimentally derived constants from Wang et al.,^18^ summarized in Table 1. Wang’s kinetics were selected over the classical Pinsent et al. correlations due to their explicit validation in high-pH regimes (pH > 12) and concentrated ionic solutions, which better align with the potassium hydroxide solvent conditions in this study. The backward reaction constants were calculated from the governing chemical equilibria across a range of reactant/product concentrations. The Aspen Plus model integrated these kinetics to simulate the process and estimate capture efficiency across varying temperatures and solvent concentrations.

**Table 1:** Arrhenius Parameters for CO_2_ Absorption Kinetics (Wang et al.,^18^)

**reaction**	*k*_0_ (m^–1^ s^–1^)	*E*_a_ (kJ mol^–1^)
${\mathrm{CO}}_{2}+{\mathrm{OH}}^{-}\to {{\mathrm{HCO}}_{3}}^{-}$	2 × 10^14^	64
${\mathrm{CO}}_{2}+2H_{2}O\to {{\mathrm{HCO}}_{3}}^{-}+H_{3}O^{+}$	1.2 × 10^11^	81

### 3.2 Electrochemical Stack Modeling

The electrochemical system was modeled in Aspen Custom Modeler, which can be embedded in Aspen Plus flowsheets as a unit operation, allowing direct integration with the absorber. The stack was modeled as *N* cell pairs in parallel, and for each cell pair a mass balance was solved. The energy consumption of each electrochemical cell pair was evaluated by considering the cell voltage and the total current passing through the cell. To simplify the modeling process and enhance tractability, the following assumptions were made:

- Well-mixed system: No concentration gradients were assumed across the length or width of the electrochemical cell. The well-mixed assumption applies specifically to the acidifying and alkaline compartments, where concentrations were considered uniform.
- Equilibrium chemistry: The chemical reactions in the system were modeled as equilibrium reactions, neglecting mass transfer limitations.
- Ideal gas assumption: The gas phase, including CO_2_ and other gases, was assumed to behave as an ideal gas, simplifying the calculations of gas-phase concentrations and partial pressures.
- Vapor–liquid equilibrium (VLE) modeled via Henry’s law: The relationship between the concentration of CO_2_ in the liquid and gas phases was described using Henry’s law, assuming that the solubility of CO_2_ in the liquid phase follows this equilibrium.
- Isothermal operation: The system was assumed to operate at a constant temperature, conductive heating effects were neglected.

#### 3.2.1 Mass and Energy Balance

A detailed mass and energy balance was conducted for the electrochemical regeneration process. The potassium ion flux across the membrane (*J*_K+_) was calculated based on the current density and the Faraday efficiency of the cell. The CO_2_ desorption rate was determined by solving a mass balance over the acidifying compartment (Figure 3), considering the proton flux and assuming negligible CO_2_ solubility in water.

**Figure 3:**
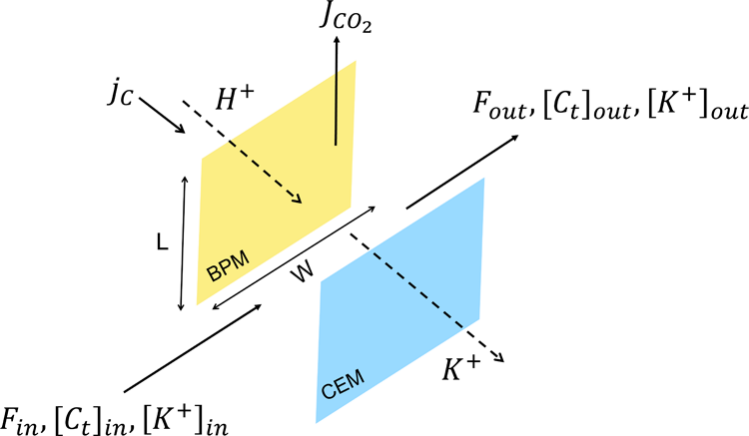
Potassium ions (K^+^) migrate across the membrane (dimensions defined by width *W* and length *L*). The schematic highlights the current density (*j*_C_), CO_2_ production rate (*J*CO_2_), and changes in carbon concentration in the acidifying compartments.

The balance equation for the potassium ion (K^+^) under steady-state conditions, assuming negligible water transport, can be expressed as follows. The net flux of potassium across the membrane is determined by the difference in inlet and outlet concentrations, scaled by the volumetric flow rate and membrane area. This flux relationship is given by

$$J_{K^{+}}=\frac{Q}{A}\left(c_{\mathrm{in}}-c_{\mathrm{out}}\right)$$ 12

where *Q* is the volumetric flow rate, *A* is the membrane area, and *c*_in_, *c*_out_ represent the inlet and outlet potassium concentrations, respectively. The dissolved inorganic carbon (DIC), defined as the sum of carbonate ([CO_3_^2–^]), bicarbonate ([HCO_3_^–^]), and dissolved CO_2_ ([CO_2_^*^]), remains constant across pH variations. This mass conservation law governs the speciation of carbonates in the acidifying compartment, which contains potassium ions ([K^+^]), hydroxide ions ([OH^–^]), protons ([H^+^]), and carbonate species. Charge balance in the acidifying compartment requires:

$$2\left[{\mathrm{CO}}_{3}^{2-}\right]+\left[{\mathrm{HCO}}_{3}^{-}\right]+\left[{\mathrm{OH}}^{-}\right]=\left[K^{+}\right]+\left[H^{+}\right]$$ 13

This ensures the sum of anionic charges equals the sum of cationic charges.

#### 3.2.2 Ion Transport and Nernst–Planck Equations

The ion flux across the membrane is governed by Nernst–Planck equations:

$$J_{K^{+}}=-D_{K^{+}}\left(\frac{dc_{K^{+}}}{dx}+\frac{z_{K^{+}}F}{RT}c_{K^{+}}\frac{d\varphi }{dx}\right)$$ 14

$$J_{H^{+}}=-D_{H^{+}}\left(\frac{dc_{H^{+}}}{dx}+\frac{z_{H^{+}}F}{RT}c_{H^{+}}\frac{d\varphi }{dx}\right)$$ 15

where *J*_K^+^_, *J*_H^+^_: Fluxes of K^+^ and H^+^ (mol m^–2^s^–1^), *D*_K^+^_, *D*_H^+^_: Diffusivities of K^+^ and H^+^ (m^2^s^–1^), *c*_K^+^_, *c*_H^+^_: Concentrations of K^+^ and H^+^ (mol m^–3^), *z*_K^+^_ = *z*_H^+^_ = +1: Charge numbers, *F*: Faraday constant (96,485 C mol^–1^), *R*: Gas constant (8.314 J mol^–1^K^–1^), *T*: Temperature (K), φ: Dimensional electric potential (V), *x*: Position inside the membrane (m).

For computational simplicity, these equations are normalized by introducing $\phi =\frac{F}{RT}\varphi$ . The normalized equations become:

$$J_{K^{+}}=-Dc_{x}\left(\frac{df_{K^{+}}}{dx}+f_{K^{+}}\frac{d\phi }{dx}\right)$$ 16

$$J_{H^{+}}=-\alpha Dc_{x}\left(\frac{df_{H^{+}}}{dx}+f_{H^{+}}\frac{d\phi }{dx}\right)$$ 17

where: $\alpha =\frac{D_{H^{+}}}{D_{K^{+}}}$ : Diffusivity ratio, ϕ: Normalized potential (dimensionless), *f*_K^+^_, *f*_H^+^_: Normalized concentration fractions, defined as

$$f_{K^{+}}=\frac{c_{K^{+}}}{c_{x}},\;f_{H^{+}}=\frac{c_{H^{+}}}{c_{x}}\;(0\leq f_{i}\leq 1)$$

*c*_*x*_: Fixed charge density of the CEM, with

$$c_{K^{+}}+c_{H^{+}}=c_{x}$$

The ionic current density is derived from the total ion flux:

$$j_{c}=F(J_{K^{+}}+J_{H^{+}})$$ 18

where *j*_c_ is the current density (A m^–2^).

The total ion flux density *J*_tot_, resolving the interplay between K^+^ and H^+^, is

$$J_{\mathrm{tot}}=\frac{\left(J_{K^{+}}-\frac{Dc_{x}}{\delta }d_{H^{+}}\right)\left[1+\left(\alpha -1\right)f_{H^{+},m}\right]}{1-f_{H^{+},m}}-\frac{Dc_{x}}{\delta }\left(\alpha -1\right)d_{H^{+}}$$ 19

where δ: Membrane thickness (m), *d*_H^+^_: H^+^ concentration gradient (dimensionless), *f*_H^+^,m_: H^+^ fraction at the membrane/alkaline interface.

Equation 19 combines the normalized Nernst–Planck fluxes (eqs 16 and 17) with electroneutrality (*f*_K^+^_ + *f*_H^+^_ = 1). This equation provides a detailed expression for the total ion current density, considering both the proton and potassium ion fluxes, and the interplay between diffusion and electric fields across the membrane.

#### 3.2.3 Numerical Solution of Governing Equations

The model consists of 24 governing equations: (1) nine equilibrium reactions describing chemical processes such as carbon dioxide hydration, bicarbonate and carbonate ion dissociation, and water self-ionization, applied to the acidifying and alkaline compartments and input stream; (2) three electroneutrality conditions enforcing charge balance in each compartment; (3) nine mass balances ensuring conservation of species including dissolved carbon dioxide, hydrogen ions, potassium ions, bicarbonate ions, and carbonate ions across the system; and (4) three flux-current relationships derived from ion transport principles and total current density definitions. These equations form a coupled system solved numerically, with additional constraints for numerical stability.^19^ Newton’s method was used with a convergence tolerance of 10^–6^ for all residuals. The solution workflow integrated all compartments (acidifying, membrane, alkaline) into a single computational framework:

- System initialization**:** The upper and lower limits, along with initial guesses for the variables, were defined.
- Full-system assembly**:** Governing equations for each compartment—including proton generation in the acidifying compartment, Nernst–Planck transport across the membrane, and CO_2_ absorption/hydroxide equilibria in the alkaline compartment—were assembled into a unified system.
- Newton iterations**:** The 24-equation system was solved iteratively using Newton’s method. At each step, residuals for charge conservation, reaction equilibria, ion fluxes, and electroneutrality were evaluated. The Jacobian matrix, accounting for interdependencies between variables, was updated numerically to ensure robustness. The system is governed by coupled equations across three compartments:
$$F(x)=\left[\begin{array}{l} F_{1}=K_{w}-[H^{+}][{\mathrm{OH}}^{-}]\\ F_{2}=K_{1}-[H^{+}][{\mathrm{HCO}}_{3}^{-}]\\ \vdots \\ F_{24}=I_{\mathrm{applied}}-F\sum z_{i}J_{i} \end{array}\right],x=\left[\begin{array}{l} \left[H^{+}\right]\\ \phi \\ \left[{\mathrm{CO}}_{3}^{2-}\right]\\ \vdots \end{array}\right]$$ 20
  1. **Jacobian Construction** via numerical differentiation:
$$J_{mn}=\frac{\partial F_{m}}{\partial x_{n}}\approx \frac{F_{m}(x+\zeta e_{n})-F_{m}(x)}{\zeta },\;\zeta =10^{-8}$$ 21
  2. **Linear System Solution**:
$$J^{\left(k\right)}\Delta x^{\left(k\right)}=-F(x^{\left(k\right)})\;(\mathrm{Solved}\mathrm{via}\mathrm{LU}\mathrm{decomposition})$$ 22
  3. **Variable Update** with damping:
$$x^{\left(k+1\right)}=x^{\left(k\right)}+\alpha \Delta x^{\left(k\right)},\;\alpha =\{\begin{array}{ll} 0.7, & k\leq 5\\ 1.0, & k>5 \end{array}$$ 23
  4. **Convergence Check**:
$$\underset{i}{\max}\left|F_{i}\right|<\epsilon_{\mathrm{abs}}=10^{-6}$$ 24
$$\underset{j}{\max}\left|\frac{\Delta x_{j}}{x_{j}}\right|<\epsilon_{\mathrm{rel}}=10^{-8}$$ 25
- **Convergence criteria:** Iterations continued until residuals for all variables (e.g., H^+^, K^+^, OH^–^ concentrations, membrane potential, carbonate speciation) fell below 10^–6^, ensuring self-consistency across the coupled physics.
- **Postprocessing:** Converged solutions were parsed to extract compartment-specific outputs (e.g., CO_2_ capture rate, pH gradients, current efficiency).

Constraints (e.g., thermodynamic equilibrium constants, mass conservation, etc.) were enforced via algebraic relationships within the equation system. While the model is structured modularly to represent distinct physical domains (acidifying/membrane/alkaline), the fully coupled solving approach ensures that cross-compartment interactions—such as proton flux influencing alkaline pH and vice versa—are captured inherently. A summary of key parameters, such as membrane conductivity values used in the model, is provided in Table 3 for reference.

### 3.3 Potential Losses

In CO_2_ electrochemical cells, several types of potential losses reduce their operational efficiency. Understanding these losses is essential for estimating the energy consumption of CO_2_ desorption and designing better-performing systems. These losses include ohmic losses, pH-related losses, membrane-related losses, and overpotential losses.

#### 3.3.1 Ohmic Losses

Ohmic losses (η_ohm_) arise from the resistance encountered by ions and electrons as they move through the electrochemical cell. In this study, we only focus on the ohmic losses in the electrolyte, across the acidifying and cathode compartments, because the resistance in these regions dominates due to the relatively low conductivity of the electrolyte compared to the highly conductive electrodes. This is typically expressed as a function of the conductivity and thickness of the respective sections as shown by the following equation:

$$\eta_{\mathrm{ohm}}=J_{c}\cdot \left(\frac{d_{a}}{\sigma_{a}}+\frac{d_{c}}{\sigma_{c}}\right)$$ 26

where η_ohm_ is the ohmic overpotential (V), *d*_a_ is the thickness of the acidifying compartment (m), *d*_c_ is the thickness of the alkaline compartment (m), σ_a_ is the conductivity of the acidifying electrolyte (S/m), σ_c_ is the conductivity of the alkaline electrolyte (S/m).

The overall electrolyte conductivity was approximated by summing the contributions of each individual electrolyte component *i*, as described by Kohlrausch’s Law:

$$\sigma_{\mathrm{electrolyte}}=\underset{i}{\sum }C_{i}\sigma_{i}$$ 27

where σ_electrolyte_ is the total conductivity of the electrolyte (S/m), *C*_*i*_ is the concentration of the *i*-th electrolyte component (mol/m^3^), σ_*i*_ is the conductivity of the *i*-th electrolyte component (S/m).

The magnitude of ohmic losses increases proportionally with current density and can lead to significant energy inefficiencies, particularly at higher currents. To mitigate these losses, high-conductivity materials and thinner compartments are often employed. Additionally, cell design strategies focus on optimizing the distribution of current and minimizing contact resistances. The electrolyte conductivity values and compartment thicknesses are provided as inputs from the model.

#### 3.3.2 pH-Related Losses

pH-related losses in CO_2_ electrochemical cells arise due to the equilibrium potential difference caused by pH gradients between the cathode and anode compartments. While pH gradients are generally minimized in typical electrochemical processes, in CO_2_ desorption, a significant pH difference is intentionally created to facilitate the release of CO_2_ from the solution. This is a deliberate strategy to drive the desorption process, which differentiates it from electroreduction systems that typically aim for a smaller pH difference.

The equilibrium potential loss due to the pH gradient between the anode and cathode is governed by the Nernst equation and is expressed as

$$\eta_{\mathrm{pH}}=0.059\left({\mathrm{pH}}_{\mathrm{cathode}}-{\mathrm{pH}}_{\mathrm{anode}}\right)$$ 28

where: η_pH_ is the potential loss due to the pH gradient (V), pH_cathode_ and pH_anode_ are the pH values at the cathode and anode, respectively.

Here, the pH at the anode is intentionally fixed at 0.27. This low value corresponds to the pH of a fully saturated Nafion 117 membrane (CEM), where there is a high concentration of H^+^ ions. Conversely, the pH at the cathode is determined by the bulk phase, which is influenced by the varying concentrations of hydroxide.

#### 3.3.3 Membrane-Related Losses

Membrane-related losses are primarily attributed to the Donnan potential, which arises from the selective permeability of the CEM. This potential difference occurs due to the concentration gradient of potassium ions (K^+^) across the membrane and can be calculated as follows:

$$\eta_{\mathrm{mem}}=\frac{RT}{F}\ln\;\frac{C_{K^{+},c}}{C_{K^{+},a}}$$ 29

where *R* is the universal gas constant, *T* is the temperature, *F* is the Faraday constant, and *C*_K^+^,c_ and *C*_K^+^,a_ are the potassium ion concentrations at the cathode and anode.

#### 3.3.4 BPM Losses

BPMs introduce additional overpotential due to the energy required for water dissociation at their junction, which involves splitting water molecules into H^+^ and OH^–^ ions. In addition to this, BPMs are also subject to counterion crossover, particularly at low current densities, where the diffusion of counterions across the membrane becomes a significant contributor to potential losses. In fact, at lower current densities, counterion crossover may represent the largest potential loss, overshadowing the overpotential associated with water dissociation alone.

The activation overpotential, η_act_, further complicates the energy losses in electrochemical cells with BPMs. η_act_ represents the additional potential required to overcome the activation energy barrier for the electrochemical reaction to proceed at both the anode and the cathode. This overpotential is necessary to initiate the electrochemical reaction and overcome barriers due to slow electron transfer kinetics or sluggish ion movement at the electrode surfaces. The Butler–Volmer equation mathematically models the relationship between current density and activation overpotential, as shown in eq 30:

$$j_{c}=j_{\mathrm{co}}\left(\exp\left(\frac{\alpha nF\eta_{\mathrm{act}}}{RT}\right)-\exp\left(-\frac{(1-\alpha )nF\eta_{\mathrm{act}}}{RT}\right)\right)$$ 30

Here, α is the electron transfer coefficient, *n* is the number of electrons transferred, and *j*_co_ is the exchange current density, which is given by

$$j_{\mathrm{co}}=\gamma \exp\left(\frac{-E_{\mathrm{act}}}{RT}\right)$$ 31

where γ is the preexponential factor and *E*_act_ is the activation energy. However, no existing literature specifically provides the constants for a system similar to the one discussed in this study.

In contrast, Ulleberg^20^ proposed an empirical model that offers a basic form of a BPM’s polarization curve for a given operating temperature, emphasizing that polarization curves are sensitive to temperature. This model allows for the fitting of individual contributing overpotentials. Amores et al.^21^ expanded on this by incorporating new parameters, including the electrode-membrane distance (*d*). Their model, shown in eq 32, includes the reversible, ohmic, and activation overpotentials:

$$V_{\mathrm{cell}}=V_{\mathrm{rev}}+[(r_{1}+d_{1})+r_{2}T+d_{2}p]j_{c}+s\log\left[\left(t_{1}+\frac{t_{2}}{T}+\frac{t_{3}}{T^{2}}\right)j_{c}+1\right]$$ 32

where

$$\eta_{\mathrm{BPM}}=\eta_{\mathrm{act}}=s\log\left[\left(t_{1}+\frac{t_{2}}{T}+\frac{t_{3}}{T^{2}}\right)j_{c}+1\right]$$ 33

is the activation overpotential term, with *s* (V) and *t* (m^2^/A) parameters that account for the correlation of activation overpotential. Bui et al.^22^ provided a measured polarization curve for a 1 M KHCO_3_ electrochemical system, which is similar to the system used in ConsenCUS. Using their experimental data, we fitted the polarization curve to eq 32 to obtain the relevant parameters for calculating the activation overpotential in the ConsenCUS stacks. Figure 4 shows the fitted polarization curve, which represents the total voltage as a result of the combination of Ohmic, reversible, and overpotential losses around the BPM. This resulted in the following parametrization for the activation overpotential:

$$\eta_{\mathrm{BPM}}=0.2065\times \log\left[\left(0.1277+\frac{9.318\times 10^{-5}}{T}+\frac{3.369\times 10^{-7}}{T^{2}}\right)j_{c}+1\right]$$ 34

**Figure 4:**
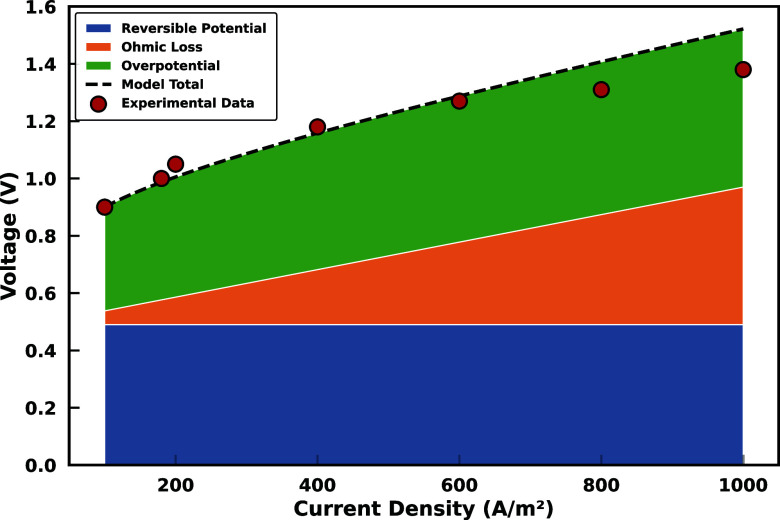
Fitted polarization curve for the 1 M KHCO_3_ electrochemical system. The experimental data are obtained from Bui et al.^22^

#### 3.3.5 Total Potential Losses

The total potential loss is calculated as the sum of individual contributions from distinct sources:

$$\eta_{\mathrm{total}}=\eta_{\mathrm{Ohm}}+\eta_{\mathrm{pH}}+\eta_{\mathrm{mem}}+\eta_{\mathrm{BPM}}$$ 35

The first three terms (η_Ohm_, η_pH_, and η_mem_) correspond to losses in the centrally positioned CEM (Figure 2), representing bulk ohmic resistance, pH gradients near the CEM interface, and membrane resistance, respectively. The final term (η_BPM_) represents the activation overpotential for water dissociation at the BPM, derived from its polarization curve to capture the dominant voltage barrier for water splitting (eq 34). Each type of loss contributes uniquely to the overall inefficiency of the cell, but they are interconnected. Improvements in one area can often reduce losses in another. For instance, reducing ohmic losses by using thinner, more conductive membranes can also decrease overpotentials by enhancing reaction kinetics at the electrode surfaces.

### 3.4 Model Outputs

#### 3.4.1 Load Ratio

The K^+^ load ratio (*L*_K^+^_) is a dimensionless metric that evaluates the efficiency of potassium ion transport within an electrochemical system. Mathematically, it is defined as

$$L_{K^{+}}=\frac{j_{C}\cdot A}{C_{K^{+}}\cdot Q\cdot F}$$ 36

where *j*_C_ is the current density (A/m^2^), *A* is the electrode area (m^2^), *C*_K^+^_ is the concentration of K^+^ ions in the feed (mol/m^3^), *Q* is the volumetric flow rate (m^3^/s), *F* is the Faraday constant (96,485 C/mol).

An *L*_K^+^_ value of 1 signifies that the cell current is precisely adequate to transport all potassium ions supplied to the cathode, highlighting an ideal transport balance.

#### 3.4.2 CO_2_ Flux and Capture Efficiency

The flux of CO_2_ produced, representing the amount of CO_2_ generated per unit membrane area (i.e., production rate), is calculated as

$$J_{{\mathrm{CO}}_{2}}=\frac{Q}{A_{a}}\cdot C_{{\mathrm{CO}}_{2},\mathrm{out}}$$ 37

where *Q* is the rich solvent flow rate (m^3^/s), *A*_a_ is the membrane area (m^2^), *C*_CO_2_,out_ is the CO_2_ concentration in the outlet stream (mol/m^3^).

The dissolved CO_2_ concentration is capped by its temperature-dependent solubility limit, governed by a modified Henry’s law:

$$C_{{\mathrm{CO}}_{2},\mathrm{sat}}=P_{{\mathrm{CO}}_{2}}\cdot K_{H}\cdot \exp\left(\frac{d\ln\;K_{H}}{d(1/T)}\left(\frac{1}{T}-\frac{1}{298.15}\right)\right)$$ 38

where *P*_CO_2__ is the CO_2_ partial pressure (bar), *K*_H_ is the Henry’s constant at 298.15 K,

-
$\frac{d\ln\;K_{H}}{d\left(1/T\right)}$ is derived from the van’t Hoff equation.

If *C*_CO_2_,out_ > *C*_CO_2_,sat_, the excess CO_2_ partitions into the gas phase.

It should be mentioned that the electrical conductivity of the acidic solution (σ_solution_) is adjusted to account for CO_2_ bubble formation. Due to CO_2_’s low solubility, gas bubbles form and reduce conductivity, modeled via:

$$\sigma_{\mathrm{solution}}=\sigma_{0}\cdot \frac{1-B\gamma \phi_{{\mathrm{CO}}_{2}}}{1+AB\phi_{{\mathrm{CO}}_{2}}}$$ 39

$$\gamma =1+\frac{\left(1-\phi_{m}\right)}{\phi_{m}^{2}}\phi_{{\mathrm{CO}}_{2}}$$ 40

where σ_0_ is the bubble-free conductivity, *A* = 1.5, *B* = 0.67 are shape-dependent parameters for gas bubbles,^23^ ϕ_CO_2__ is the CO_2_ bubble volume fraction, ϕ_m_ = 0.637 is the maximum packing gas fraction.

Gas bubbles are assumed to be nonconductive, consistent with the approach applied in electrochemical systems by Sabatino et al.^24^

The CO_2_ capture efficiency (capture rate) in the absorber was evaluated by

$$\eta_{{\mathrm{CO}}_{2},\mathrm{capture}}\left(\%\right)=\frac{y_{{\mathrm{CO}}_{2},\mathrm{in}}-y_{{\mathrm{CO}}_{2},\mathrm{out}}}{y_{{\mathrm{CO}}_{2},\mathrm{in}}}\cdot 100$$ 41

where *y*_CO_2_,in_ and *y*_CO_2_,out_ are the mole fractions of CO_2_ in the gas stream at the absorber’s inlet and outlet, respectively.

#### 3.4.3 Specific Energy Consumption and CO_2_ Production Rate

In the electrochemical regeneration process, the specific energy consumption (SEEC) was calculated as

$$\mathrm{SEEC}=\frac{j_{C}\cdot A_{a}\cdot V_{\mathrm{total}}}{J{˙}_{{\mathrm{CO}}_{2}}}$$ 42

where *V*_total_ is the stack voltage (V), *j*_C_ is the current density (A/m^2^), *A*_a_ is the active membrane area (m^2^), *J̇*_CO_2__ is the CO_2_ gas flow rate produced in the regeneration cell (mol/s).

### 3.5 Results and Discussion

#### 3.5.1 Model Validation

The model was validated against experimental data from Kuntke et al.^25^ for CO_2_ production rates and SEEC under systematically varied conditions: current densities, flow rates and carbon loadings. The experimental conditions are summarized in Table 2 (for further details, please refer to ref 25.

**Table 2:** Experimental Parameters for Electrochemical Regeneration of CO_2_-Loaded KOH^a^

parameter	range
current density (mA/cm^2^)	15–100
flow rate (mL/min)	3–83
voltage (V)	4.8–18.1
CO_2_ production rate (mol/m^2^ s)	reported in Figure 5
SEEC (MJ/kg CO_2_)	reported in Figure 5

a Data adapted from Kuntke et al.,^25^ available under a CC BY 4.0 license.

Figure 5 compares modeled and experimental results, with data points color-coded by current density. Proximity to the diagonal parity line indicates agreement, while deviations highlight model limitations. The results show that the model effectively predicts CO_2_ production rates with a reasonable degree of accuracy, as indicated by a low margin of error. However, deviations at high current densities reveal inaccuracies likely due to mass transport limitations and nonlinear effects. These observations align with challenges common in electrochemical systems, where simulations often align closely with experimental results at low current densities but diverge at higher currents due to limitations such as reactant/product transport inefficiencies and structural changes in electrodes.^26–28^ The modeled CO_2_ production rates also show an offset compared to the experimental CO_2_ production rates. This offset may stem from both the model implementation as well as the experiment: the model is simplified and excludes activities and rigorous γ–ϕ vapor liquid equilibria, possibly leading to overestimation of the CO_2_ production. Meanwhile, we observed in our laboratory measurements that small gas bubbles formed in the liquid do not necessarily evolve spontaneously, suggesting they are technically in the gas phase, but need an additional driving force to move from nucleation to bubble growth. SEEC discrepancies are pronounced across all regimes (Figure 5.b). At low current densities, the model underestimates SEEC by a noticeable margin, as observed in several experiments. This stems from two factors: (1) the model neglects electrode activation overpotentials (it refers to the energy barrier for electron transfer at the electrode surfaces) and parasitic reactions (e.g., hydrogen evolution reaction (HER), and oxygen evolution reactions (OER)), which dominate energy losses at low currents. Notably, the model does include BPM overpotential from water dissociation but excludes electrode-specific kinetic losses; and (2) experimental ohmic losses (e.g., contact resistances) are not fully captured.

**Figure 5:**
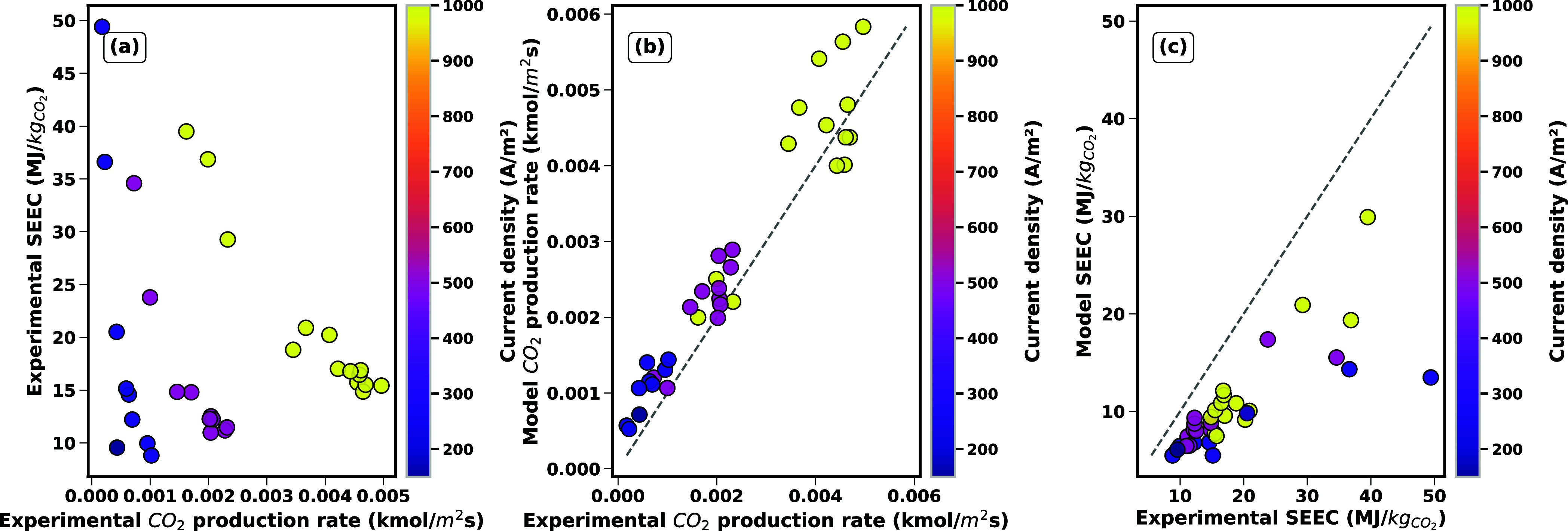
Experimental measurements of SEEC and desorbed CO_2_ (mol/m^2^ s) at different current densities, predicted desorbed CO_2_ (mol/m^2^ s) for electrochemical regeneration of CO_2_-loaded aqueous KOH, and predicted SEEC (MJ/kg CO_2_) for electrochemical regeneration. Data points are color-coded by current density.

#### 3.5.2 Pilot Plant Modeling

The integrated Aspen Plus/ACM model was used to simulate the ConsenCUS pilot plant. The specifications for the pilot plant components, including detailed parameters of the absorber and electrochemical cell, are provided in Table 3. This section presents a selection of these results to exemplify the use of the model, analyzing KPIs in response to variations in operational parameters. Specifically, we examine the impact of changes in solvent flow rate, gas flow rate, and current density across three case studies, each characterized by different CO_2_ content. The results illustrate how these operational adjustments influence system efficiency and performance, providing insight into process behavior under varying conditions.

**Table 3:** Pilot Plant Specifications for CO_2_ Absorption and Electrochemical Regeneration

component	parameter	value/range	notes
absorber	column type	packed-bed	designed for CO_2_ capture from gas streams.
	packed height	9 m	
	diameter	0.6 m	
	packing type	Pall ring	plastic
	packing diameter	15.875 mm	
	packing void fraction	0.9	
	packing specific surface area	300 m^2^/m^3^	
	solvent	aqueous KOH	0.05 M K_2_SO_4_ was added as a conductivity promoter.
electrochemical cell	configuration	bipolar membrane electrodialysis	stack with alternating cation-exchange membranes.
	membrane type	BPM-CEM	facilitates K^+^ transport
	membrane (CEM) area	44 cm × 44 cm	
	number of stacks	2	
	number of cell per stack	126	
	fixed charge density (α)	2	
	membrane thickness (δ)	4.8 × 10^–4^ m	
	potassium diffusivity	1.96 × 10^–9^ m^2^/s	
	proton diffusivity	9.31 × 10^–9^ m^2^/s	
	anode (cathode) thickness (d)	0.25 × 10^–2^ m	
	current density	10–150 mA/cm^2^	

In Figure 6, the gas flow rate was set to 200 kg/h, while the solvent flow rates varied from 400, to 600 and 1000 kg/h, resulting in an L/G ratio range of 2 to 5. Optimizing the liquid-to-gas (L/G) ratio is important for balancing CO_2_ removal efficiency and energy consumption in CO_2_ capture systems.

**Figure 6:**
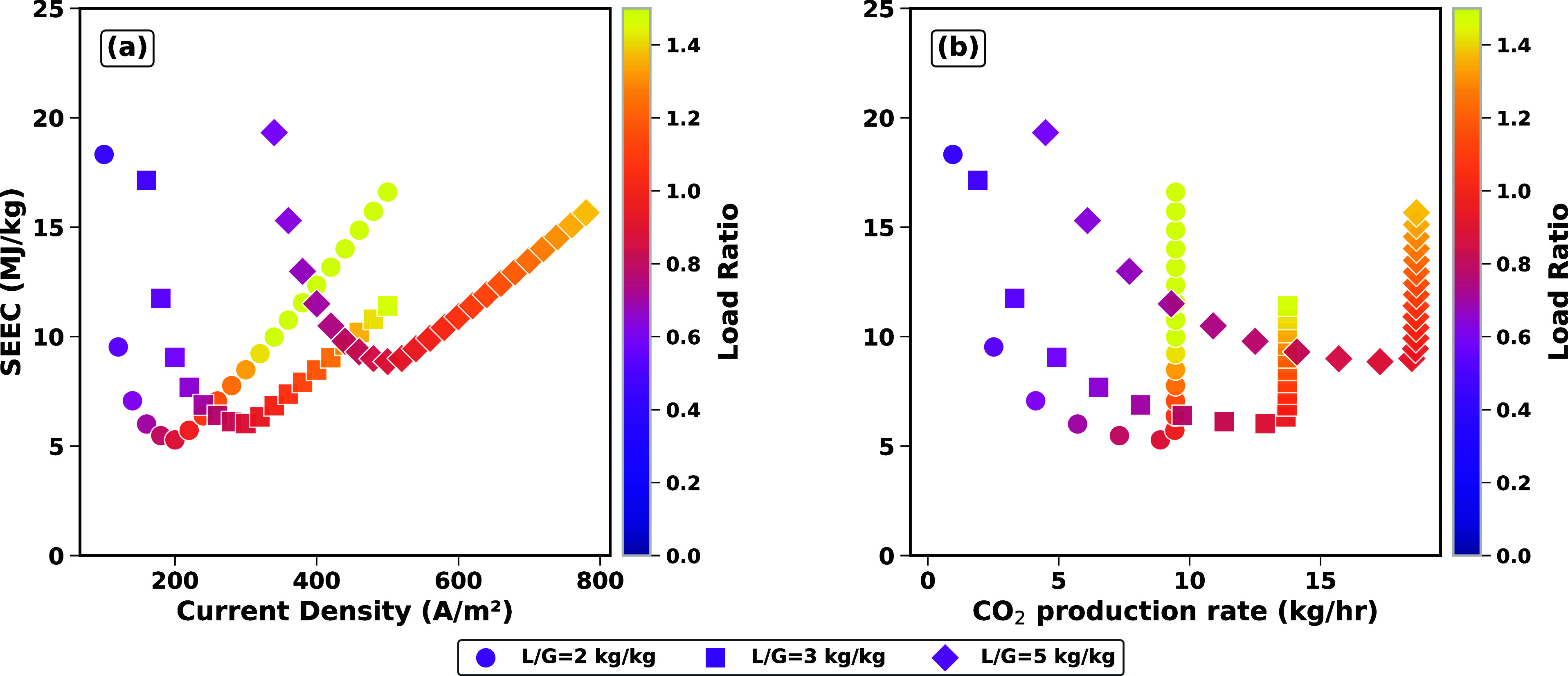
Simulation results for a case study with 3.5% CO_2_ content in flue gas (CHP) and a constant flue gas flow rate of 200 kg/h, with varying lean solvent flow rates highlighting the performance across different L/G ratios and load ratios: (a) SEEC versus current density; and (b) SEEC versus CO_2_ production rate. Colors indicate the resulting load ratio.

First, Figure 6a shows the relationship between current density (A/m^2^), specific energy consumption (SEEC, MJ/kg), and L/G ratio (−), with higher L/G ratios correlating with increased energy consumption. Specifically, while a higher L/G ratio improves CO_2_ capture by increasing the amount of solvent available for absorption, it also requires more energy for solvent regeneration. The SEEC increases with both current density and L/G ratio, highlighting the trade-off between CO_2_ capture efficiency and energy requirements. At higher L/G ratios (L/G = 3 and 5), the system demands higher current densities to regenerate the solvent, leading to greater energy consumption, as indicated by the steep rise in SEEC. The capture rate approaches 100% for L/G = 5 (circles) compared to just below 90% for L/G = 3 (squares) and just over 60% for L/G = 2 (triangles). Figure 6b show that indeed at lower L/G ratios and using the current solvent, the maximum solvent loading is fully met, imposing a hard ceiling to the amounts of CO2 captured and regenerated. Second, Figure 6a shows that for each L/G ratio there is a minimum achievable energy consumption, just like for thermally regenerated systems, where there is an optimum between CO_2_ evolution/regeneration and energy input, i.e., losses in the electrochemical stacks. The color coding highlights that these optima appear at different load ratios, i.e., around 0.86, 0.83, and 0.84, for L/G = 2, 3, and 5, respectively. These first results emphasize the importance of identifying an optimal L/G ratio that maximizes CO_2_ capture while minimizing energy consumption. The optimal balance ensures effective CO_2_ removal without excessive energy costs, as higher load ratios tend to improve capture efficiency but at the expense of increased energy input.

Figure 7 demonstrates the inverse relationship between *rich solvent loading* (dissolved inorganic carbon concentration over solvent concentration, here, K^+^) and SEEC for the three different case studies: CHP, Magnesite, and Cement. A lower liquid-to-gas (L/G) ratio enhances the rich loading by concentrating CO_2_ in less solvent, thereby reducing the energy required for solvent regeneration (lower SEEC), as observed in CHP’s performance (minimum SEEC of 3.5 MJ/kg at 0.58 rich loading). This aligns with findings by Michailos and Gibbins,^29^ who note that lower L/G ratios improve solvent efficiency but require careful optimization to avoid operational limits such as solvent saturation. Conversely, higher L/G ratios dilute CO_2_ absorption, increasing regeneration energy demands (Andreasen,^30^), as seen in the CHP higher baseline SEEC. Balancing these factors is critical to maintaining higher capture efficiency while minimizing energy costs and maximizing solvent utilization. The CO_2_ content in the flue gas significantly influences the SEEC and rich loading. Cement outperforms Magnesite and CHP due to its ability to load the solvent to a value of 0.78 with minimal SEEC, owing to the larger CO_2_ driving force at, especially, the bottom of the absorber. In contrast, Magnesite and CHP exhibit higher SEEC values at lower rich loadings (0.5–0.67), resulting from a smaller CO_2_ driving force. Practically, optimizing the L/G ratio for each material is essential: lower ratios favor energy efficiency but at lower capture rates, while higher ratios lead to higher capture rates while providing some operational margin at the expense of energy costs.

**Figure 7:**
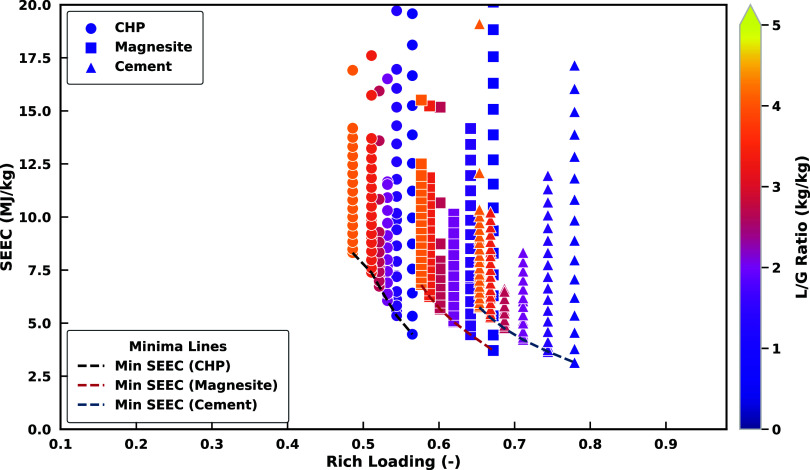
Simulation results of SEEC dependence on rich solvent carbon loading and CO_2_ content in flue gas across varying current densities and lean solvent flow rates.

The optimal K^+^ load ratios for these cases (*L*_K^+^_ = 0.8 – 0.9) achieve a balance between maximizing CO_2_ production and minimizing ohmic resistance, ensuring minimal energy consumption across all case studies (Figure 8). At this ratio, sufficient K^+^ ion availability enables efficient current density for CO_2_ desorption while mitigating ionic congestion, thereby reducing ohmic losses. This equilibrium optimizes the trade-off between reactant utilization (enhancing CO_2_ generation) and ion mobility (improving carbon removal efficiency). However, at higher load ratios (*L*_K^+^_ > 1.0), the CO_2_ production rate diminishes due to limited bicarbonate availability and increased ohmic resistance caused by ionic crowding. Here, the H^+^ flux dominates due to its higher mobility (α > 1), outcompeting K^+^ for transport pathways. This leads to nonproductive H^+^ recirculation once HCO_3_^–^ is fully converted to CO_2_, while K^+^ transport is further hindered by overcrowding. The resulting ionic congestion forces K^+^ ions to collide more frequently, competing for limited pathways and drastically slowing their movement. These collisions convert useful electrical energy into wasted heat, escalating the energy required to sustain ion flow. The predicted H^+^ and K^+^ fluxes align with Nernst–Planck kinetics under idealized assumptions (e.g., no concentration polarization or gas–liquid partitioning), representing an upper bound for flux rates. In practice, real-world systems would experience additional losses, consistent with experimental observations of ion-exchange membranes under overlimiting currents.^14^ The energy penalty at *L*_K^+^_ > 1.0 arises from the need for elevated current densities to overcome resistance, which yields diminishing CO_2_ returns and higher overall energy demands. Thus, maintaining *L*_K^+^_ < 0.9 is critical for sustaining the balance between ion transport efficiency and resistive energy losses, as earlier demonstrated by Shu et al.^14^

**Figure 8:**
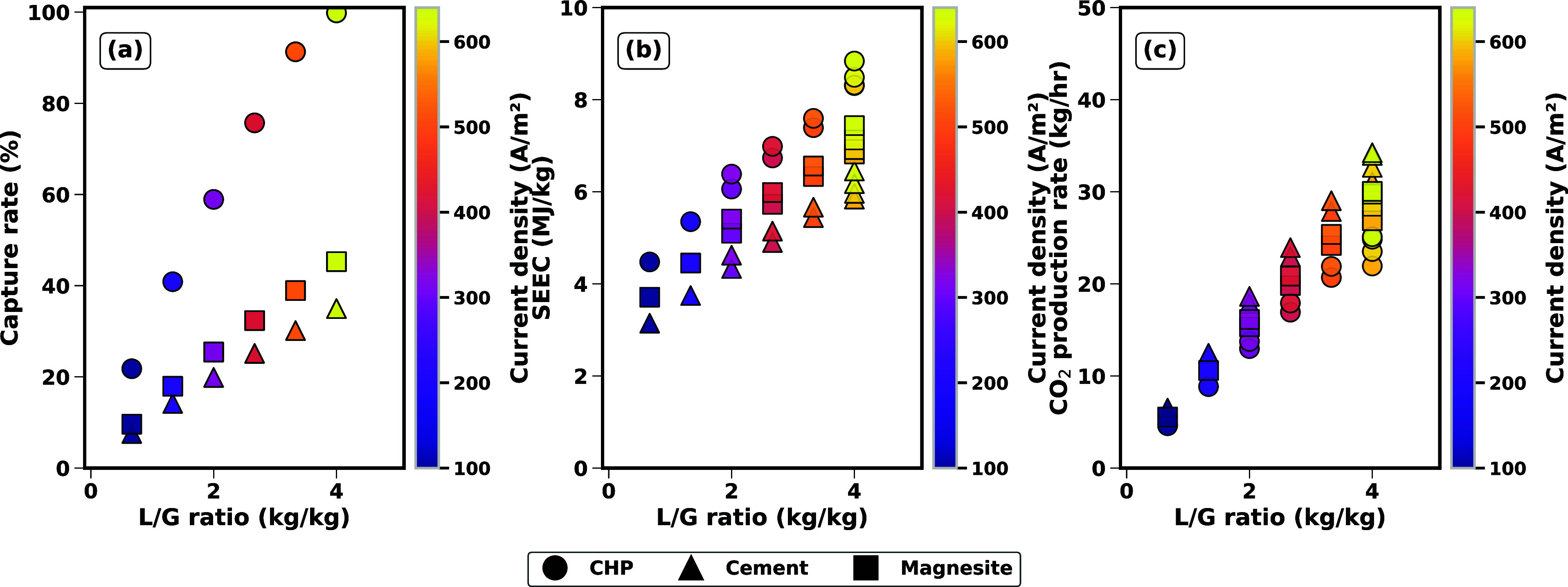
Simulation results for the best performance of the electrochemical cell (*L*_K^+^_ = 0.8 – 0.9) across all case studies at different L/G ratios and current densities: (a) capture rate; (b) SEEC; and (c) CO_2_ production rate.

Figure 8 shows the effect of varying L/G, while maintaining an optimal load ratio (0.8–0.9) for each case study. CHP, with a CO_2_ content of 3.5%, represents a scenario with low CO_2_ concentration, which results in lower CO_2_ production rates (as seen in Figure c) and higher energy demands (SEEC) to capture CO_2_ and regenerate the solvent, particularly at higher L/G ratios. Magnesite, with a moderate CO_2_ content of 9.1%, achieves a balance between CO_2_ capture efficiency and production rates, benefiting from higher capture rates at relatively lower L/G ratios. Cement, with the highest CO_2_ content of 13.5%, exhibits the most efficient performance in terms of CO_2_ production rates and capture efficiency, as its higher CO_2_ concentration reduces the energy required per unit of CO_2_ captured (lower SEEC). These trends highlight the critical role of the initial CO_2_ content in determining the energy efficiency and operational effectiveness of the electrochemical cell, with higher flue gas CO_2_ concentrations favoring better overall performance at the optimal load ratio of 0.8–0.9.

The simulation results in Figure 9 illustrate the interplay between liquid-to-gas (L/G) ratios and load ratios (color-coded, 0–2) on CO_2_ capture performance at a fixed current density of 400 A/m^2^. CHP shows the highest capture efficiency, nearing 100% at high L/G ratios (4.0). Magnesite and cement face challenges to achieve high capture efficiency, peaking below 40% at high L/G ratios and dropping to 20% at lower ratios. SEEC tracks capture performance in the opposite direction: The high capture rates of CHP result in increased energy costs, while cement achieves better energy efficiency at intermediate capture rate. It can be seen from the results that there is a minimum in SEEC (or a maximum in CO_2_ production rates) at an optimal L/G ratio of approximately 2.7, corresponding to a load ratio of 0.8–0.9, despite diverging capture efficiencies. At this operating point, SEEC drops below 5 MJ/kg CO_2_ for the Cement case study, and CO_2_ production peaks at 20 kg/h for Magnesite and 23 kg/h for Cement — significantly outperforming their baseline values. CHP, while maintaining a capture rate of approximately 75% at this ratio, requires the SEEC of 7 MJ/kg, underscoring that electrochemical constraints (fixed current density of 400 A/m^2^) govern this optimum. This suggests that operational tuning of L/G ratios and load ratios can simultaneously minimize energy costs and maximize throughput, even when capture efficiency is suboptimal.

**Figure 9:**
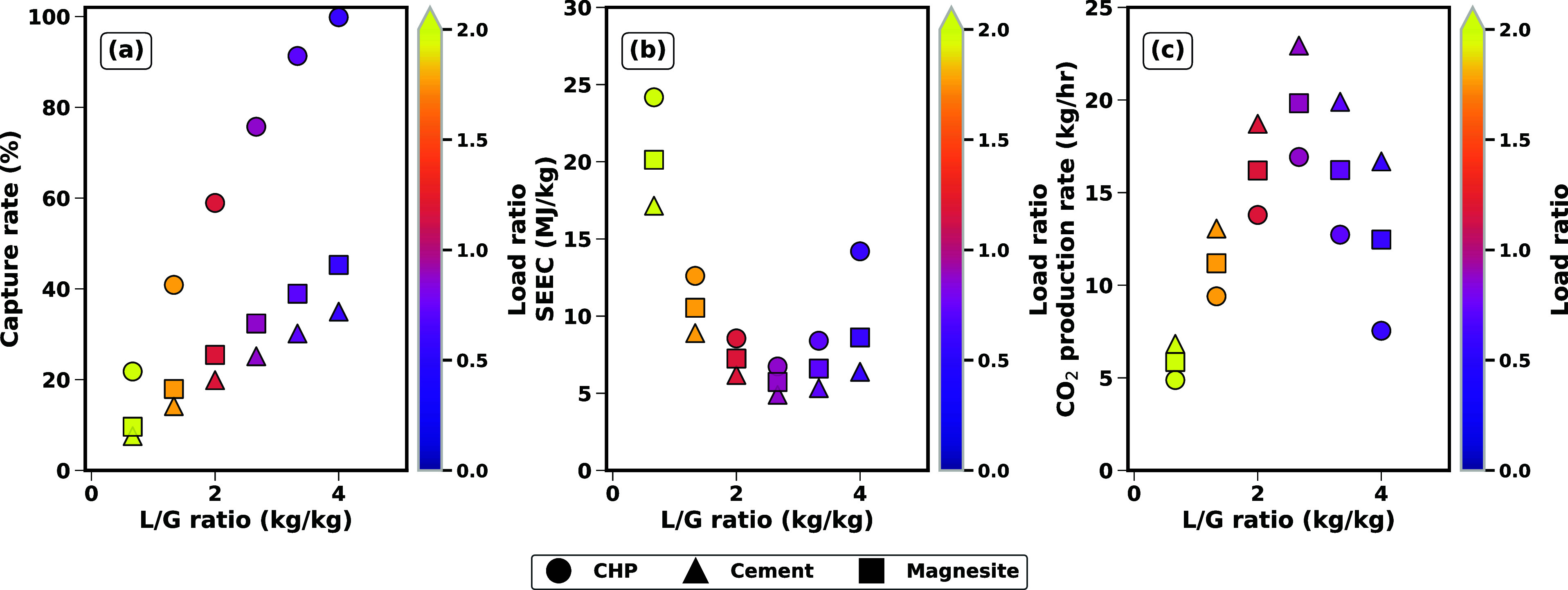
Simulation results for varying L/G ratios at a fixed current density of 400 A/m^2^ across all case studies: (a) capture rate; (b) SEEC; and (c) CO_2_ production rate.

## 4 Conclusions

We here presented an integrated CO_2_ absorption - electrochemical regeneration model, developed in Aspen Plus (absorption and overall process flowsheet) and Aspen Custom Modeler (electrochemical stack, embedded in overall process flowsheet). The electrochemical model is equilibrium-based and simultaneously solves a system of equations coupling chemical equilibria in acidifying and alkaline compartments, cation transport over a cation exchange membrane, and CO_2_ evolution. The electrochemical CO_2_ regeneration model was validated against experimental data, showing good agreement in CO_2_ production rates at low current densities (15 mA cm^–2^) but deviating at higher currents due to unmodeled mass transport effects and simplifications (e.g., simplified vapor–liquid equilibria). SEEC discrepancies, particularly at low currents, arose from omitted activation losses and idealized ohmic resistances. Pilot plant simulations for CO_2_ capture from three industrial flue gas types revealed trade-offs: increasing the L/G ratio (2–5) enhanced capture rates (60–100%) but raised SEEC by up to 300%, while higher flue gas CO_2_ content (3.5–13.5%) reduced energy demands. Optimal K^+^ loading (*L*_K^+^_ = 0.8 – 0.9) balanced CO_2_ production and ohmic losses, whereas higher loads (*L*_K^+^_ > 1.0) increased resistance. Industrial scenarios highlighted divergent priorities: CHP achieved near-complete CO_2_ capture (100%, 60 MJ kg^–1^) while cement case study prioritized throughput (7 kg h^–1^, 10 MJ kg^–1^). The study underscores the need for tailored parameter optimization and improved modeling of transport dynamics and energy losses.

## Nomenclature List

**Symbols**
*A*: membrane/electrode area (m^2^)
*A*_a_: active membrane area (m^2^)
*a*: ratio of proton-to-potassium ion diffusivities
*C*: concentration (mol/m^3^)
*C*_CO_2_,out_: outlet CO_2_ concentration (mol/m^3^)
*c*_in_, *c*_out_: inlet/outlet potassium ion concentrations (mol/m^3^)
*c*_*x*_: total cation concentration in membrane (mol/m^3^)
*D*: diffusivity coefficient (m^2^/s)
*d*: thickness (m); *d*_*a*_, *d*_*c*_ = anode/cathode compartment thickness
*E*_a_: activation energy (kJ/mol)
*F*: Faraday constant (96485 C/mol)
*f*_K^+^_, *f*_H^+^_: fractional concentration of K^+^/H^+^ in membrane
*J*: flux (mol/m^2^/s); *J*_K^+^_, *J*_H^+^_ = K^+^/H^+^ flux
*J*_CO_2__: CO_2_ production rate (mol/s)
*j*_c_: current density (A/m^2^)
*k*_0_: pre-exponential factor in Arrhenius equation (s^–1^)
*K*_1_, *K*_2_: equilibrium constants for CO_2_ hydration/bicarbonate dissociation
*K*_*w*_: water dissociation constant
*L*_K^+^_: potassium ion load ratio (dimensionless)
*N*: number of cell pairs
*Q*: volumetric flow rate (m^3^/s)
*R*: universal gas constant (8.314 J/mol · K)
*T*: temperature (K)
*V*_total_: total stack voltage (V)
α: charge transfer coefficient
δ: membrane thickness (m)
η_ohm_: ohmic overpotential (V)
η_pH_: pH-related potential loss (V)
η_mem_: membrane Donnan potential loss (V)
η_BPM_: bipolar membrane activation overpotential (V)
η_total_: total potential loss (V)
σ: conductivity (S/m); σ_*a*_, σ_*c*_ = anode/cathode conductivity
Φ: electric potential (V)
**Acronyms**
BPM: bipolar membrane
CEM: cation exchange membrane
CHP: combined heat and power
DIC: dissolved inorganic carbon ([CO_2_^*^] + [HCO_3_^–^] + [CO_3_^2–^])
E-NRTL: electrolyte non-random two-liquid (thermodynamic model)
SEEC: specific energy consumption (MJ/kg_CO2_)
VLE: vapor–liquid equilibrium
**Chemical Species**
CO_2_*: dissolved CO_2_ (aqueous)
HCO_3_^–^: bicarbonate ion
CO_3_^2–^: carbonate ion
H^+^: proton
OH^–^: hydroxide ion
K^+^: potassium ion
**Units**
Concentrations: mol/m^3^
Current density: A/m^2^
Fluxes: mol/m^2^/s
Potential: V
**Notes**
Subscripts: in (inlet), out (outlet), a (acidifying), c (cathode).
Brackets denote molar concentrations: (e.g., [H^+^]).
